# The Diagnostic Value of the Pleural Fluid C-Reactive Protein in Parapneumonic Effusions

**DOI:** 10.1155/2016/7539780

**Published:** 2016-04-18

**Authors:** Shimon Izhakian, Walter G. Wasser, Benjamin D. Fox, Baruch Vainshelboim, Mordechai R. Kramer

**Affiliations:** ^1^The Pulmonary Institute, Rabin Medical Center, Beilinson Hospital, 49100 Petah Tikva, Israel; ^2^The Sackler Faculty of Medicine, Tel Aviv University, 69978 Tel Aviv, Israel; ^3^2nd Department of Medicine, Semmelweis University, Budapest 1088, Hungary; ^4^Mayanei HaYeshua Medical Center, 51544 Bnei Brak, Israel; ^5^Rambam Health Care Campus, 3109601 Haifa, Israel

## Abstract

*Purpose*. The aim of this study was to evaluate the sensitivity of pleural C-reactive protein (CRP) biomarker levels in identifying parapneumonic effusions.* Methods*. A single-center, retrospective review of 244 patients diagnosed with pleural effusions was initiated among patients at the Rabin Medical Center, Petah Tikva, Israel, between January 2011 and December 2013. The patients were categorized into 4 groups according to their type of pleural effusion as follows: heart failure, malignant, post-lung transplantation, and parapneumonic effusion.* Results*. The pleural CRP levels significantly differentiated the four groups (*p* < 0.001) with the following means: parapneumonic effusion, 5.38 ± 4.85 mg/dL; lung transplant, 2.77 ± 2.66 mg/dL; malignancy, 1.19 ± 1.51 mg/dL; and heart failure, 0.57 ± 0.81 mg/dL. The pleural fluid CRP cut-off value for differentiating among parapneumonic effusions and the other 3 groups was 1.38 mg/dL. The sensitivity, specificity, positive predictive value, and negative predictive value were 84.2%, 71.5%, 37%, and 95%, respectively. A backward logistic regression model selected CRP as the single predictor of parapneumonic effusion (OR = 1.59, 95% CI = 1.37–1.89).* Conclusions*. Pleural fluid CRP levels can be used to distinguish between parapneumonic effusions and other types of exudative effusions. CRP levels < 0.64 mg/dL are likely to indicate a pleural effusion from congestive heart failure, whereas levels ≥ 1.38 mg/dL are suggestive of an infectious etiology.

## 1. Introduction

A parapneumonic effusion is a pleural effusion associated with lung infection [1]. Early in the course of parapneumonic effusion, the pleura becomes inflamed with leakage of cellular elements, protein, and fluid into the pleural space, forming the effusion. Subsequent bacterial invasion results in a frank empyema, the presence of which often requires thoracentesis. A delay in the diagnosis and initiation of proper therapy for infectious effusions leads to increases in the complication rate. These delays are more common in patients with coexisting heart failure or malignancy [2, 3].

The existence of inadequate diagnostic criteria is a major reason for a delay in diagnosis. Pleural leukocyte counts, effusion cells, differential counts, and Light's criteria do not reliably identify an infectious etiology [3]. Although a pleural pH < 7.20 and pleural glucose < 60 mg/dL are indications for pleural drainage, these thresholds are not sufficiently sensitive [4]. Moreover, other conditions, such as malignancy, tuberculosis, rheumatoid pleurisy, and lupus pleuritis, can cause pleural fluid acidosis or low pleural glucose, demonstrating that these indicators lack specificity for infection. [5]. Although a pleural white blood cell count >50,000 cells/*μ*L may help accurately diagnose parapneumonic effusions, pleural white cell counts more often range between 10,000 and 50,000. As a result, they are not diagnostic [6]. Although microbiologic studies provide definitive evidence of infection, positive cultures are seen in only 60% of parapneumonic effusions, and there is often a prolonged time to culture positivity [7, 8].

Because the classic pleural biochemistry testing lacks both sensitivity and specificity, the development of a novel pleural biomarker for infection has been an area of active investigation. Procalcitonin, interferon-*γ*, carcinoembryonic antigen, interleukin-6, tumour necrosis factor-*α*, and soluble triggering receptor expressed on myeloid cells-1 (STREM-1) have been evaluated for their utility in distinguishing empyema from other types of effusions, but they are not sensitive for detection [8–10].

CRP is an acute phase protein that is synthesized by the liver in response to various stimuli [11]. The induction of CRP synthesis in the liver is triggered by the production of IL-6 and TNF-*α* by local pleural cells [12, 13]. The pleural fluid CRP levels are likely to reflect the serum levels because the presence of CRP in the pleural fluid may be due to increased diffusion from the blood as a result of inflamed capillary leakage [12, 13].

Pleural CRP has recently been proposed as a specific biomarker for the differential diagnosis of pleural effusions and reportedly exhibits higher sensitivity and specificity than serum CRP [14]. CRP can be considered a good candidate due to its 1000-fold elevation in response to infection and the positive correlation between the serum and pleural CRP levels [15, 16]. However, few studies with limited samples sets have been published on this topic [9, 10, 17–19].

The aim of this study was to evaluate the sensitivity and specificity of pleural CRP levels in diagnosing parapneumonic effusions and the role of pleural CRP levels in distinguishing exudative effusions from transudative effusions. In this retrospective study, we evaluated the utility of pleural CRP as a novel biomarker of infection in the pleural space.

## 2. Materials and Methods

A retrospective, single-cohort study of clinically significant pleural effusions was performed at the Rabin Medical Center in Petah Tikva, Israel. The inclusion criteria in the study were as follows: (1) ambulatory patients who were under outpatient observation at the Rabin Medical Center Pulmonary Institute and were diagnosed with a new pleural effusion and (2) hospitalized patients who were referred for pulmonary consultation from internal medical services and received a diagnostic thoracentesis.

The study population consisted of 244 individuals who were treated at our institution between January 2011 and December 2013. The diagnoses were divided into five categories on the basis of the underlying disease.

The diagnosis of malignant effusion was made when malignant cells were found on pleural fluid cytologic examination or in a biopsy specimen.

The pleural effusion was considered parapneumonic when it was associated with acute febrile illness with purulent sputum, pulmonary infiltrate responsive to antibiotic treatment or when a microorganism was identified in the pleural fluid. Empyema was defined as a thick, purulent appearance of parapneumonic effusion.

Tuberculous pleural effusion was diagnosed based on positive cultures for mycobacterium tuberculosis or when the pleural biopsy specimen revealed typical epithelioid cell granulomas.

The effusion was attributed to congestive heart failure (CHF) in individuals with findings of an enlarged heart, radiographic pulmonary venous congestion, and peripheral edema responding to diuretic treatment in the absence of malignancy or pulmonary infiltrates associated with an inflammatory process.

The diagnosis of post-lung transplantation pleural effusion was made in patients who had undergone recent lung transplantation and lacked evidence of malignancy, infection, or rejection.

This study was approved by the Rabin Medical Center Institutional Review Board. Written informed consent was not required in this observational, retrospective study as per guidelines of the Rabin Medical Center Institutional Review Board.

## 3. Laboratory Studies

Pleural fluid samples were obtained with thoracentesis before treatment soon after the diagnosis of pleural effusion. Samples were analyzed for total differential cell counts, CRP, glucose, total protein, lactate dehydrogenase (LDH), pH, amylase, and cholesterol. Additionally, cytologic examination and bacterial cultures using blood agar, chocolate agar, and MacConkey agar, Lowenstein medium, and Mycobacterium Growth Indicator Tubes (BACT MGIT 960, Becton Dickinson, USA) were routinely obtained for all pleural fluid samples. All specimens were analyzed for mycobacteria using Ziehl-Neelsen stain.

The supernatant was obtained by centrifugation at 300 rpm for 15 min and stored at 20°C until being assayed. The clinicians who performed the laboratory studies were blinded to the clinical diagnosis of the pleural effusion.

CRP analysis was performed on a Beckman Coulter AU 2700 analyzer using a particle-enhanced immune-turbidimetric method and latex particles coated with monoclonal anti-CRP antibodies. The test is linear within a concentration range of 0.008–8 mg/dL. The CRP reference range values were 0–0.5 mg/dL.

## 4. Statistical Analysis

Statistical analyses were performed with the Chi-square test for categorical variables and ANOVA test for continuous variables, as appropriate; *p* values < 0.05 were considered significant.

To determine the variables that were most significantly associated with parapneumonic effusion, we included all pleural parameters that were traditionally used to indicate the type of effusion (PH, LDH, glucose, neutrophils, and CRP) in a backward stepwise logistic regression.

To evaluate the diagnostic performance of CRP, as a marker for differentiating between parapneumonic effusions and other pleural effusions, receiver operator characteristics (ROC) analysis was performed for all significant differences between groups. ROC curves were generated by plotting the sensitivity against 1 − specificity, and the area under the curve (AUC) with 95% confidence intervals (95% CI) was calculated. The optimum cut-off point based on the ROC analysis was established by selecting the value that provides the greatest sum of the sensitivity and specificity, that is, the point closest to the upper left point of the ROC plot. For the optimum cut-off point provided by each ROC analysis, the sensitivity, specificity, positive predictive value (PPV), and negative predictive value (NPV) were calculated using standard formulas. To calculate the ROC curves and AUCs, we used SAS version 9.2 software (SAS Institute Inc., Cary, NC, USA).

## 5. Results

Of the 244 patients classified as having pleural effusions, 180 (73.7%) were diagnosed with exudative effusion, 44 (18%) were diagnosed with transudative effusion, and 20 (8.1%) were excluded from the study due to lack of definitive diagnosis. The exudative effusion group was further divided into the following three subgroups according to the diagnosis: 119 (53.1%), malignant effusion; 38 (16.9%), parapneumonic effusion; and 23 (10.2%), lung transplant recipients (Table 1). Tuberculous pleural effusion was not diagnosed in any patient.

**Figure 1:**
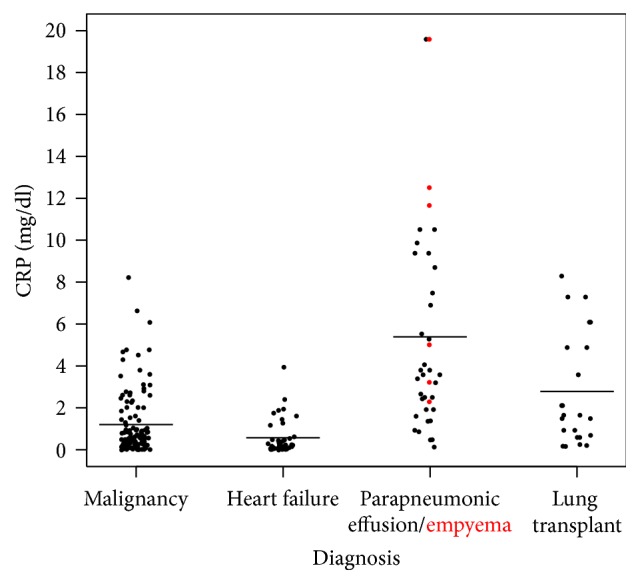
Pleural fluid CRP levels in effusions secondary to pneumonia, malignancy, post-lung transplantation, and heart failure. Each point represents one pleural fluid sample. The red points represent patients with empyema.

The pleural CRP levels differed significantly among all four groups (*p* < 0.001). The mean values from the highest to the lowest were as follows: parapneumonic effusion (5.38 ± 4.85 mg/dL), lung transplant (2.77 ± 2.66 mg/dL), malignancy (1.19 ± 1.51 mg/dL), and heart failure (0.57 ± 0.81 mg/dL) (Figure 1).

A backward logistic regression model selected CRP as the only predictor of parapneumonic effusion (OR = 1.59, 95% CI = 1.37–1.89, and *p* < 0.0001).

**Figure 2:**
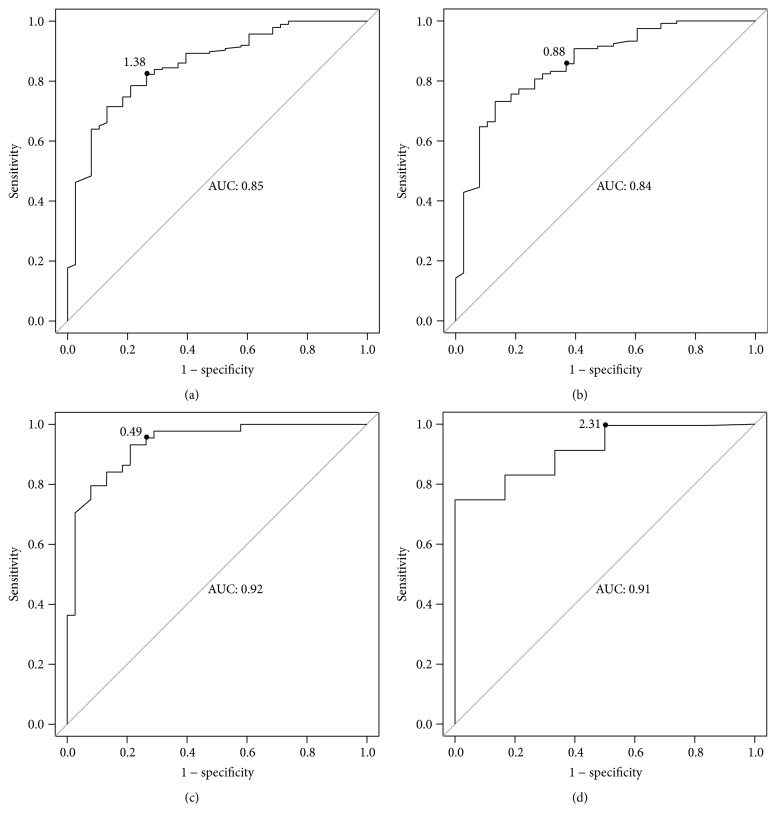
Receiver operator characteristic (ROC) analysis curves of pleural fluid CRP levels for differentiating between different effusion types. (a) ROC curve of CRP levels for differentiating parapneumonic pleural effusions from other types of pleural effusions such as malignant, heart failure, and post-lung transplantation effusions. (b) ROC curve of CRP levels for differentiating between parapneumonic and malignant effusions. (c) ROC curve of CRP for differentiating between parapneumonic and heart failure pleural effusions. (d) ROC curve of CRP for differentiating empyema from other types of effusion such as malignant, heart failure, and uncomplicated parapneumonic pleural effusions.

To determine the efficiency of pleural fluid CRP measurement in distinguishing parapneumonic effusion from the other 3 groups, we used ROC analysis (Figure 2(a)). A CRP cut-off value of 1.38 mg/dL yielded 84.2% sensitivity, 71.5% specificity, 37.6% positive predicted value, and 95.6% negative predicted value. Although the area under the curve (AUC) of the pleural CRP was as high as 0.85 (Figure 2), it was lower for the following other pleural parameters: glucose (0.54), pH (0.61), neutrophils (0.59), and LDH (0.57). As a result, we could not calculate their optimal cut-off values.

CRP was a good marker for distinguishing parapneumonic effusion from post-lung transplantation effusion (1.93 mg/dL cut-off value, 75% sensitivity, and 56% specificity), malignant effusion (0.88 mg/dL cut-off value, 87% sensitivity, and 64% specificity; Figure 2(b)), and heart failure effusion (0.49 mg/dL cut-of value, 93% sensitivity, and 72% specificity; Figure 2(c)).

CRP was a moderately good marker for differentiating between empyema and other types of effusions (2.31 mg/dL cut-off value, 83.3% sensitivity, 74.7% specificity, 8.3% positive predicted value, and 99.3% negative predicted value; Figure 2(d)).

We also determined the efficiency of pleural fluid CRP measurements in distinguishing between heart failure effusion and the other 3 groups (Table 2). Using a cut-off value of 0.64 mg/dL, CRP exhibited 79.5% sensitivity, 59.4% specificity, 32.4% PPV, and 92.4% NPV.

Pleural white blood cells counts were significantly different among all four groups (*p* = 0.003) with the following means: 9.13 ± 21.82 mg/dL (lung transplant), 2.49 ± 7.56 mg/dL (malignancy), 1.59 ± 1.84 (parapneumonic effusion), and 0.81 ± 1.05 mg/dL (heart failure).

Pleural neutrophil differentials were also significantly different among all four groups (*p* = 0.01): 30.5 ± 26.3% (parapneumonic effusion), 22.33 ± 24.53% (lung transplant), 21.31 ± 15.48% (malignancy), and 17.86 ± 11.86% (heart failure).

## 6. Discussion 

The present study provides evidence for the utility of the pleural fluid CRP measurement in diagnosing parapneumonic effusions. Early recognition of this diagnosis prevents possible adverse consequences from an untreated infection of the pleural cavity. We found that the pleural CRP levels were higher in parapneumonic effusion than in other effusion types, with a cut-off value of >1.38 mg/dL. At this cut-off level, we found a low PPV but very high NPV, which implies a modest utility in confirming the diagnosis but a powerful tool for excluding it. The same is true with empyema; due to its high NPV, a cut-off value below 2.31 can theoretically decrease the necessity of pleural drainage for complicated effusions. Our results are consistent with other studies that reported higher CRP levels in parapneumonic effusions among the exudative categories, which exhibit a range of cut-off values of 3 to 9 mg/dL [2, 9, 10, 14, 18]. The differences in the absolute cut-off values can be attributed to the different analysis methods used for measuring CRP. Moreover, when the underlying cause of a pleural effusion is obscure, a high pleural CRP level combined with pleural neutrophil predominance, lower pleural glucose, and lower pleural pH may shift the diagnosis towards an infectious etiology.

This study corroborates and amplifies previous investigations. Pleural CRP levels are reportedly higher in parapneumonic effusions than in other types of exudates [2, 14, 18]. Evaluation of the pleural CRP levels is a useful test for differentiating between complicated and uncomplicated parapneumonic effusions [9, 17] and between acute and chronic inflammation [18]. Kapisyzi et al. [14] observed that the sensitivity of pleural CRP levels was higher than serum CRP levels in distinguishing transudative effusions from exudative effusions as well as malignant effusions from benign effusions. A prospective evaluation of seven biological markers in patients with different causes for exudative effusions demonstrated that CRP provides the largest AUC (0.92) for distinguishing between parapneumonic effusions and tuberculosis or malignant effusions [19]. Kriopoulos et al. and Gabhale et al. reported that pleural CRP levels provided excellent sensitivity (100%) and good specificity (79, 98.8%) at cut-off levels of 5.3 and 9.08 mg/dL, respectively, for differentiating between parapneumonic effusion and tuberculosis or malignant effusions [2, 18]. A few investigative reports have suggested that the combination of pleural fluid CRP levels with neutrophil count [9, 19] or adenosine deaminase [10] is superior to pleural CRP levels alone for predicting parapneumonic effusion.

Finally, in addition to exhibiting diagnostic value, pleural CRP levels exhibit prognostic value and can serve as a supporting tool for drainage. Porcel et al. [9] found that CRP levels >10 mg/dL were associated with complicated parapneumonic effusion and were associated with the need for pleural effusion drainage. Moreover, the combination of classical biomarkers (pleural pH < 7.2, LDH > 100 IU/dL, and glucose < 60 mg/dL) improves the accuracy of detecting parapneumonic effusion [9, 17].

We also found that WBC counts were higher in pleural effusion after transplantation than those in parapneumonic group. Moreover, in both groups, the main pleural cell differential was lymphocyte. The explanation for posttransplantation pleural lymphocytosis is disruption of lymphatic flow due to severance of allograft lung lymphatics [20].

The main strengths of this study are that it demonstrates the diagnostic value of pleural CRP measurements in a large cohort of patients with varying etiologies for pleural effusion. Second, although we used several markers of inflammation individually or in combination with CRP, only CRP as a single biomarker had the highest sensitivity and specificity in differentiating between parapneumonic effusions and other effusion types. Finally, for every thoracentesis performed in this study, the same pleural biomarker panel was collected, decreasing the probability of selection bias.

A limitation of the present study is its retrospective, single-center design. A second limitation is the inclusion requirement utilizing only hospitalized or ambulatory patients under medical observation of pulmonologists necessarily excluding individuals under the care of general physicians. A third limitation is the lack of serum CRP level data which could serve for control and comparison analysis. A fourth limitation is the inability to explore the CRP trend changing level during the progression/resolving of the parapneumonic effusion, due to the availability of a single point measurement of pleural CRP to each patient. Additionally, since the decision to initiate thoracentesis was based on the judgment of the pulmonary physician, there may have been sampling bias.

## 7. Conclusions

Pleural fluid CRP levels can be used to discriminate between parapneumonic effusions and other types of exudative effusions, which may help distinguish between exudative and transudative effusions. A CRP level >1.38 mg/dL indicates the strong possibility of a parapneumonic effusion, whereas a level <0.64 mg/dL indicates a heart failure pleural effusion. This study highlights the need for prospective studies to demonstrate the prognostic effect of pleural CRP as an effective diagnostic biomarker. If confirmed in future studies, our results support the introduction of pleural fluid CRP into clinical practice for accurate detection of patients who may benefit from the initiation of antibiotic therapy and observation for the need for chest tube drainage.

## Figures and Tables

**Table 1:** Clinical characteristics of the study population and pleural fluid parameters.

	Malignancy	Heart failure	Parapneumonic total effusions	Parapneumonic empyema only	Lung transplant	*p* value
*n*	119/244 (53.1%)	44/244 (19.6%)	38/244 (16.9%)	6/244 (2.4%)	23 (10.2%)	
Male, (%)	52.9	77.3	50	60%	15	0.02
Age, years	70.9 ± 12	76.2 ± 10.6	64 ± 17.9	59.5 ± 19.7	58.3 ± 6.4	<0.001
Amount, mL	1514.4 ± 1694.5	1380.6 ± 668.2	983.4 ± 552.6	681.6 ± 439.5	715.2 ± 259.9	=0.01
CRP level, mg/dL	1.19 ± 1.51	0.57 ± 0.81	5.38 ± 4.85	9.06 ± 6.72	2.77 ± 2.66	<0.001
WBC, K/micL	2.49 ± 7.56	0.81 ± 1.05	1.59 ± 1.84	2.2 ± 1.1	9.13 ± 21.82	=0.003
Neutrophils, %	21.31 ± 15.48	17.86 ± 11.86	30.5 ± 26.3	44.3 ± 40	22.33 ± 24.53	0.01
Lymphocyte, %	51.79 ± 23.97	51.33 ± 23.27	44.92 ± 28.19	30.1 ± 30	60.99 ± 30.09	NS
Eosinophils, %	1.38 ± 2.25	0.99 ± 1.08	1.83 ± 2.92	0.76 ± 1	0.64 ± 1.05	NS
Cholesterol, mg/dL	74.64 ± 33.58	36.9 ± 18.77	59.63 ± 33.64	69 ± 26.9	82.09 ± 37.44	<0.001
Triglyceride, mg/dL	47.92 ± 186.24	21.1 ± 16.37	34.95 ± 33.21	68.4 ± 79	33.55 ± 20.96	NS
Glucose, mg/dL	116.93 ± 51.35	133.24 ± 41.22	113.74 ± 55.27	68 ± 58.7	133.7 ± 63.75	NS
Total protein, g/dL	4.31 ± 1.06	3.07 ± 1.05	3.22 ± 1.16	3.98 ± 0.96	3.51 ± 0.84	<0.001
Amylase, U/L	100.99 ± 309.9	48.97 ± 23.52	37.36 ± 21.99	37.4 ± 25.6	35.85 ± 16.22	NS
LDH, U/L	613.31 ± 1327.75	405.21 ± 1122.89	998.39 ± 2244.33	4336.6 ± 5235.1	2337.1 ± 6176.01	0.01
pH	7.45 ± 0.13	7.47 ± 0.08	7.4 ± 0.23	7.29 ± 0.32	7.47 ± 0.24	NS

All parameters are from pleural fluids.

**Table 2:** Receiver operating characteristic curve analysis of the accuracy of biomarkers for identifying parapneumonic effusions.

Biomarker	CRP optimal cut-off value (mg/dL)	Sensitivity (%)	Specificity (%)	PPV^*∗*^ (%)	NPV^*∗∗*^ (%)	AUC^*∗∗∗*^
PE^†^ versus Mal^‡^, HF^§^, and LTx^¶^	>1.38	84.2	71.5	37.6	96.7	0.85
PE versus LTx	>1.93	75.7	56.5	71.4	61.9	0.67
PE versus Mal	>0.88	87.8	64.7	40.8	95	0.84
PE versus HF	>0.49	93.9	72.7	72	94.1	0.92
HF versus Mal, PE, and LTx	<0.64	79.5	59.4	32.4	92.2	0.76
Emp^£^ versus Mal, LTx, and uncomplicated PE	>2.31	83.3%	74.7%	8.3%	99.3%	0.7

^†^PE: parapneumonic effusions; ^‡^Mal: malignancy; ^£^Emp: empyema;^§^ HF: heart failure; ^¶^LTx: lung transplant; ^*∗*^PPV: positive predictive value; ^*∗∗*^NPV: negative predictive value; ^*∗∗∗*^AUC: area under the curve.
